# Inertial Sensor-Based Variables Are Indicators of Frailty and Adverse Post-Operative Outcomes in Cardiovascular Disease Patients

**DOI:** 10.3390/s18061792

**Published:** 2018-06-02

**Authors:** Rahul Soangra, Thurmon E. Lockhart

**Affiliations:** 1 Department of Physical Therapy, Crean College of Health and Behavioral Sciences, Chapman University, Orange, CA 92866, USA; soangra@chapman.edu; 2 School of Biological and Health Systems Engineering, Ira A. Fulton Schools of Engineering, Arizona State University, Tempe, AZ 85287, USA

**Keywords:** frailty prediction, fall risk, smartphone based assessments, adverse post-operative outcome

## Abstract

Cardiovascular disease (CVD) patients with intrinsic cardiac cause for falling have been found to be frail and submissive to morbidity and mortality as post-operative outcomes. In these older CVD patients, gait speed is conjectured by the Society of Thoracic Surgeons (STS) as an independent predictor of post-operative morbidity and mortality. However, this guideline by STS has not been studied adequately with a large sample size; rather it is based largely on expert opinions of cardiac surgeons and researchers. Although one’s gait speed is not completely associated with one’s risk of falls, gait speed is a quick robust measure to classify frail/non-frail CVD patients and undoubtedly frail individuals are more prone to falls. Thus, this study examines the effects of inertial sensor-based quick movement variability characteristics in identifying CVD patients likely to have an adverse post-operative outcome. This study establishes a relationship with gait and postural predictor variables with patient’s post-operative adverse outcomes. Accordingly, inertial sensors embedded inside smartphones are indispensable for the assessment of elderly patients in clinical environments and may be necessary for quick objective assessment. Sixteen elderly CVD patients (Age 76.1 ± 3.6 years) who were scheduled for cardiac surgery the next day were recruited for this study. Based on STS recommendation guidelines, eight of the CVD patients were classified as frail (prone to adverse outcomes with gait speed ≤ 0.833 m/s) and the other eight patients as non-frail (gait speed > 0.833 m/s). Smartphone-derived walking velocity was found to be significantly lower in frail patients than that in non-frail patients (*p* < 0.01). Mean Center of Pressure (COP) radius (*p* < 0.01), COP Area (*p* < 0.01), COP path length (*p* < 0.05) and mean COP velocity (*p* < 0.05) were found to be significantly higher in frail patients than that in the non-frail patient group. Nonlinear variability measures such as sample entropy were significantly lower in frail participants in anterior-posterior (*p* < 0.01) and resultant sway direction (*p* < 0.01) than in the non-frail group. This study identified numerous postural and movement variability parameters that offer insights into predictive inertial sensor-based variables and post-operative adverse outcomes among CVD patients. In future, smartphone-based clinical measurement systems could serve as a clinical decision support system for assessing patients quickly in the perioperative period.

## 1. Introduction

Falls [1] and frailty [2,3] in elderly patients are multifactorial [4] and are attributed to a complex interaction of intrinsic and extrinsic risk factors superimposed on normal aging process [5,6,7,8]. Patients with intrinsic cardiac cause for falling have been found to have higher mortality rate than those with non-cardiovascular or unknown causes of falls [9]. Falls in cardiovascular disease (CVD) patients are reported to be caused by underlying cardiovascular disorders or are linked to aging [10]. It remains unclear which factors are responsible for high fall risk in CVD patients, but some experts speculate that certain environments, medications, age-related changes, and diseases make a particular genotype of people vulnerable to frailty and falls in CVD patients [11,12]. This frailty phenotype is independently predictive of falls [13]. Some researchers have also linked functional limitation [14,15,16,17], poor nutritional status [14,15,18], cognitive impairment [15,19,20], depression [20,21] and loneliness [22] with cardiovascular disorders and frailty. Since both fall risk and frailty are multifactorial problems, a better understanding of the variables linked to these problems on post-operative outcomes is imperative. 

Analysis of gait and postural predictor variables that describe the underlying neuromuscular function are indispensable for the diagnosis and treatment of elderly patients and may be necessary for objectively assessing CVD patients for their post-operative outcomes. Bereft of multisystem reserves, the elderly CVD patients (particularly who are frail) are increasingly vulnerable to an array of adverse health outcomes, including sarcopenia, hospitalization, negative energy balance, exhaustion, falls, and loss of independence [23] and mortality. It is evident that the elderly with cardiovascular disorders along with a history of falling have a two-thirds chance of falling over the next year [7]. In addition to inherent fall risk in older CVD patients, there exists heterogeneity of health status and this leads to increased risk of post-operative complications [16,24], and thus surgical decision-making is challenging for clinicians. Preoperative risk assessment is essential but there is a paucity of tools for predicting operative risk. Physiologic reserve in an older adult can determine his/her resilience to recover from an operation. However, there is no standardized method of measuring physiologic reserve in older surgical patients [24]. Frailty is a marker of decreased physiologic reserves and resistance to stressors [13,25,26,27] and predicts operative risk in older surgical patients [24]. In clinical operative settings, clinicians have tried to link postoperative adverse outcomes with various components of frailty [28].

Researchers have also reported that age remains an independent risk factor even after controlling for co-morbid illnesses and functional impairment for postoperative complications [14,15,16,19,28,29]. Chronological age of a patient does not reflect his/her biological age, and elderly patients have a range of biological statuses that vary from robust to frail [11,30]. Recently, the Society of Thoracic Surgeons (STS) has conjectured gait speed in CVD patients as an independent predictor of post-operative morbidity and mortality. However, this guideline by STS is based largely on expert opinion and a single walking characteristic i.e., gait speed. Although one’s gait speed is not well associated with one’s risk of falls, gait speed remains a quick robust measure to classify frail/non-frail CVD patients and undoubtedly frail individuals are more prone to falls [31,32,33]. The objective of this study is to utilize laboratory-validated tools [34,35] to assess gait and posture-related movement variability characteristics using inertial sensors (widely used in fall-risk assessment), and apply it to a clinical setting for quick assessment of post-operative adverse outcomes in cardiovascular patients. It was hypothesized that inertial sensors can help identify a subset of patients and as such their gait and posture measures have potential to identify patients with a high probability of adverse post-operative health outcome.

## 2. Materials and Methods

Sixteen CVD patients have been included in this study (Table 1). Patients were included in the study only if they: (i) Consented to participate and were above 70 years of age (ii), were going to be operated on the next day for cardiovascular disorder (cardiac surgery), (iii) were cognitively able to follow instructions and, (iv) were able to ambulate. The patients were categorized into a frail (F) group (walking velocity ≤ 0.833 m/s) and non-frail (NF) group (walking velocity > 0.833 m/s). The sample population had five females (ID17, ID18, and ID20–22) and 11 males.

Patients scheduled for cardiac surgery and present to the Cardiac Surgery Pre-Surgical Testing (PST) area of the hospital were screened by the PST nurse to determine whether all inclusion criteria were met. If the patient was found eligible for inclusion into the study, the PST nurse requested the patient if he/she were interested in talking about the study. If the patient was interested, a consenter (registered nurse specialist) discussed the study with the patient, answered all relevant questions about the study, and obtained written consent according to the IRB.

**Figure 1:**
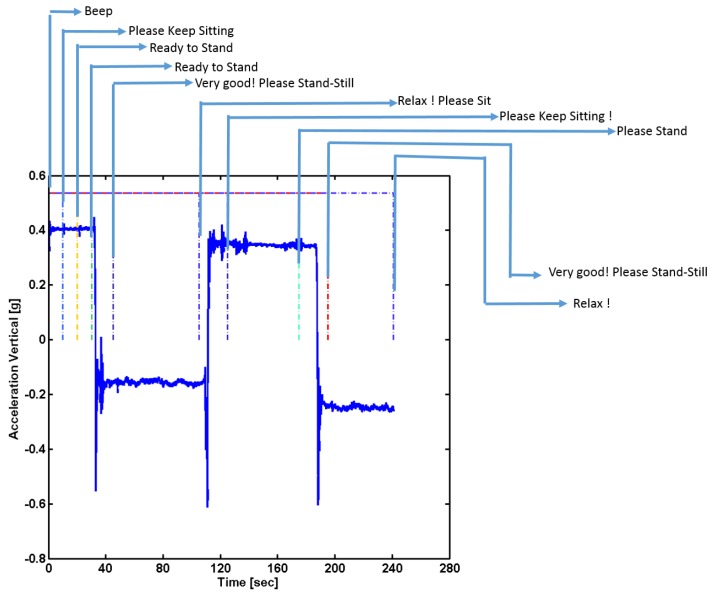
Truncation of smartphone IMU signals using temporal information of voice commands through the app.

**Figure 2:**
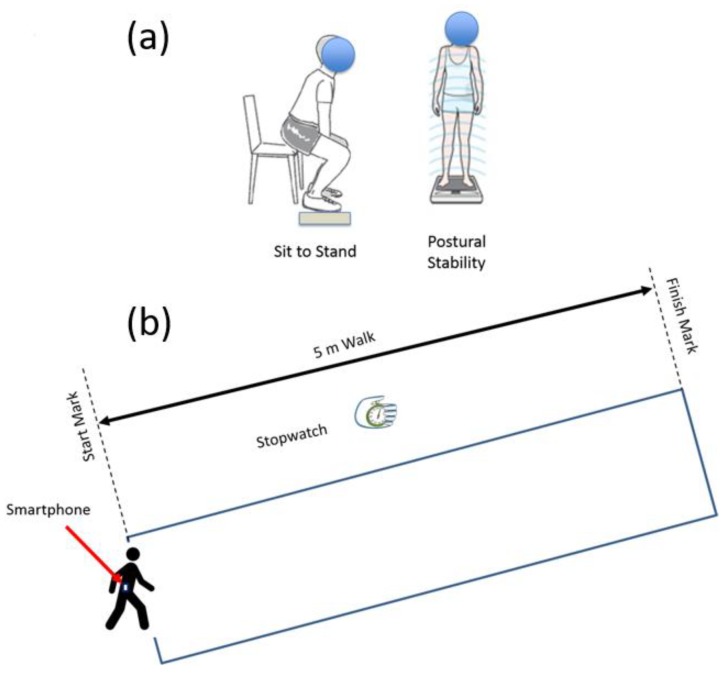
All patients (**a**) stand still for 60 s and perform sit-to-stand transitions; (**b**) walk a distance of 5 m.

Patients who met all inclusion criteria and had consented were requested to wear a waist belt and a smartphone (inside holster) was clipped to the waist belt. All the experiments were conducted in a well-lit room with an unobstructed walking area with clear floor markings at 0 m and 5 m (Figure 1). The patients were asked to rise from the chair to a standing position and follow instruction as per the voice commands of the app (Table 2). Patients were allowed to use their walking aid (cane, walker) if they needed. A standard digital stopwatch was used; the stopwatch was started with the first footfall after the 0 m line and stopped with the first footfall after the 5 m line. The walk was repeated 3 times, with sufficient time for subject recuperation between trials. Each 5 m walk time (in seconds) was recorded on the data collection form (Figure 2). The average speed for the 3 trials was calculated and was also recorded on the data collection form. The participant’s postural transition time and static postural stability was measured using the floor embedded forceplate beside the bariatric chair and smartphone-based inertial sensors. The walking speed and other gait characteristics were also determined using smartphone-based inertial sensors.

Instrumentation: In this study, we used an Apple Iphone 5 instrument (iPhone 5, Apple Inc., Cupertino, CA, USA) which contains an ultra-compact low-power high-performance 3-axis “nano” MEMS accelerometer, LIS331DLH. The LIS331DLH has user selectable full scales of ±2 g/±4 g/±8 g and it is capable of measuring accelerations with output data rates 0.5 Hz to 1 kHz. It is capable of measuring acceleration data with a data sampling rate of 1000 Hz. It also contains a low-power 3-axis angular rate sensor, L3G4200D. The L3G4200D has a full scale of ±250/±500/±2000 degrees per second and can measure angular rates at a user-selectable bandwidth. An iOS 6-based app, named as “Lockhart Monitor” (App available freely on iOS store) was designed to collect data at a sampling frequency of 50 Hz. The app was programmed in objective C language using Xcode 4 IDE (Integrated Development Environment). The data was collected from inbuilt sensors, accelerometers and gyroscopes in the smartphone and stored in it. The collected data was either transferred using cloud service/Email or by a USB cable to the computer for data analyses. Further data processing was accomplished using custom-made Matlab (MATLAB version 6.5.1, 2003, The MathWorks Inc., Natick, MA, USA) routines. The app was designed after consultations with human factors specialists and clinical requirements from registered nurse specialists. The designed mobile app consisted of a start and stop button and recorded voice instructions were provided through the app with ample rest duration inbetween each performed activity (Table 2). The signals were truncated using the temporal information of voice commands through the app (Figure 1). A portable forceplate (Bertec Corporation, FP4060-05-PT, Columbus, OH, USA) was used to measure postural stability information.

Data Analyses: The smartphone data from each participant was collected and saved in two files: (i) The three trials of 5 m gait data (ii) and postural standing and sit-to-stand transition were collected in another data file. The data was resampled to 50 Hz, using the timestamps registered by the smartphone. The continuously collected data was then truncated at intervals and the truncated signal was used for further analysis (Figure 1). The signals were filtered using a low-pass Butterworth filter with zero lag at a cut-off frequency of 6 Hz. This cut-off frequency was selected since human movements are below 3 Hz [36,37].

**Figure 3:**
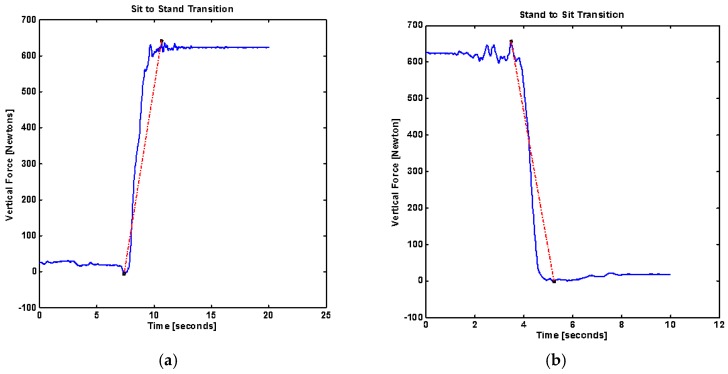
(**a**) Sit-to-stand vertical force (F_V_), ∆t is time taken from the minimum vertical force to maximum vertical force and body jerk (slope as dotted red-line); (**b**) stand-to-sit vertical force (F_V_), ∆t is time taken from the minimum vertical force to maximum vertical force and associated body jerk (slope as dotted red-line).

To quantify the postural transition several parameters were derived from the forceplate placed below the feet of the patients while standing. Body uplift jerk [Newton/s] was defined as the rate of change of force during the sit-to-stand transition. It was calculated as the slope of the line connecting the highest force to the lowest vertical force for sit-to-stand and stand-to-sit transitions (Figure 3).

(1) $$\mathrm{BUJ}=\frac{{\mathrm{dF}}_{V}}{\mathrm{dt}},$$

where F_V_ is vertical force and Δt is the transition time for sit-to-stand or stand-to-sit transition.

**Figure 4:**
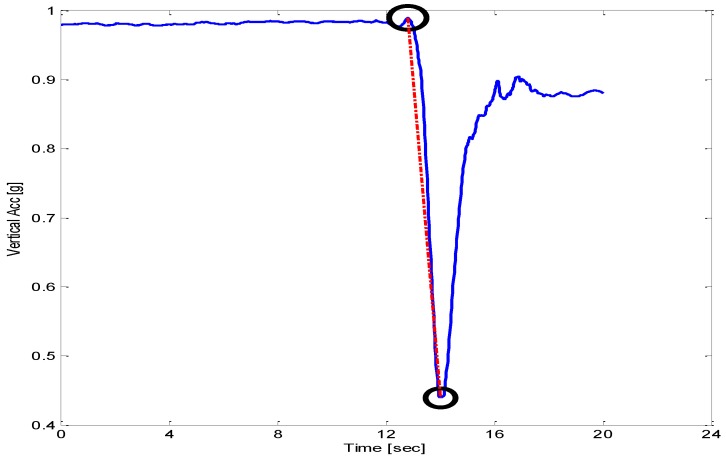
Peak flexion acceleration during sit-to-stand or stand-to-sit movements. Dotted red-line shows the slope as jerk.

Jerk [m/s^3^] was computed using accelerometer signals from steady state to maximum acceleration achieved during sit-to-stand or stand-to-sit movements. Figure 4 shows a typical signal of sit-to-stand from a smartphone accelerometer. Sway radius [mm] was calculated as the resultant of the mean of sway in AP and ML trajectories. Root Mean Square [mm] value of sway trajectory in a particular direction (AP or ML) was computed. Sway area [mm^2^] was computed using mean sway radius.

**Figure 5:**
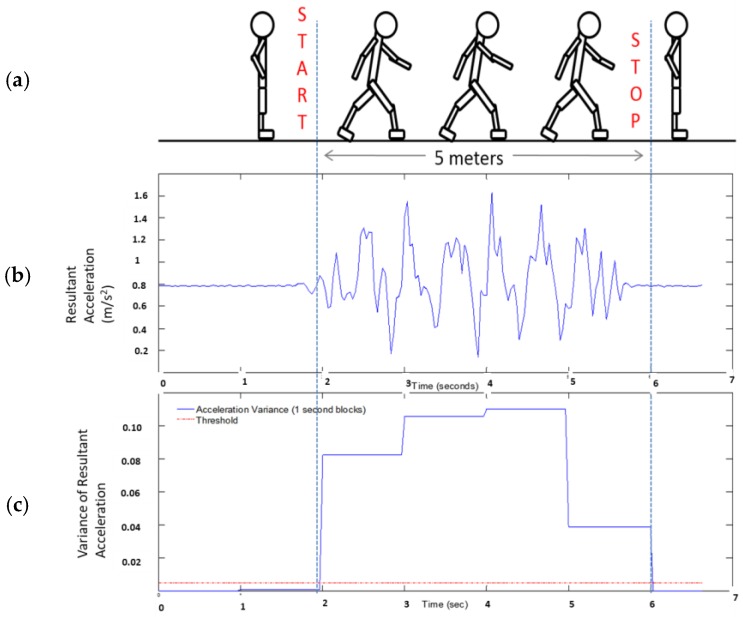
(**a**) The test starts from still-standing followed by 5 m walk and stops at still-standing as well; (**b**) resultant acceleration signals (in g-units); (**c**) moving window (0.5 s) variance of low-pass filtered resultant acceleration.

Gait speed [m/s] was computed using inertial sensors of the smartphone for a 5 m long walk [34]. Acceleration signals from three directions were used to compute resultant acceleration. The resultant acceleration signals were filtered using a 4th order dual low-pass butterworth filter with a cut-off frequency as 6 Hz. One half second moving window variance was computed and the threshold was set using initial stand still data (Figure 5) as per the experimental protocol. Once start and stop time are detected, average velocity is computed over a 5 m distance walk. Root Mean Square (RMS) is a measure of dispersion of the data relative to zero, whereas standard deviation is a measure of dispersion relative to mean. This value is an indication of average magnitude of accelerations in each direction during a complete walking trial [38].

**Figure 6:**
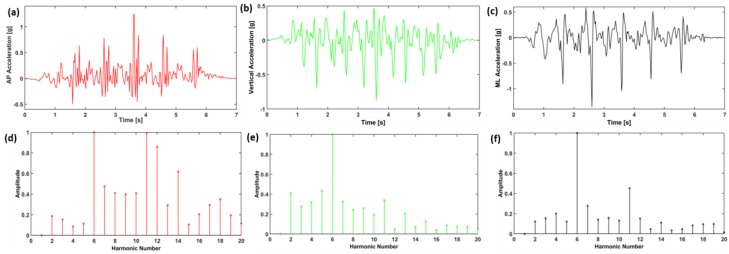
A representative acceleration signal of a patient with (**a**) acceleration in AP direction; (**b**) acceleration in vertical direction; (**c**) acceleration in ML direction; (**d**) harmonics of AP acceleration; (**e**) harmonics in vertical acceleration; (**f**) harmonics in ML acceleration for 5 m walk.

Where RMS_AP, RMS_ML, and RMS_V represent root mean square accelerations in anterior-posterior, medial-lateral and vertical directions, respectively (Table 3). RMS is statistical measure of magnitude of acceleration in each direction. RMSR represents the ratio between RMS in each direction and the RMS vector magnitude (RMST). RMSR is apparently the RMS normalized by the RMST. Harmonic ratio was described by Gage [39] and Smidt [40], to provide an indication of smoothness and rhythm of acceleration patterns. The harmonic ratio proposed by Gage is based on the premise that a stable rhythmic gait pattern should consist of acceleration patterns that repeat in multiples of two. Those which do not repeat in multiples of two are out of phase accelerations and therefore manifest as irregular accelerations during walking. The harmonic content of acceleration signal is evaluated in each direction using stride frequency as the fundamental frequency component. The acceleration signals that are in phase (even harmonics) are compared to components out of phase (odd harmonics) using finite Fourier series (Figure 6). The harmonic ratio is calculated by dividing the sum of amplitudes of the first 10 even harmonics by the first 10 odd harmonics for AP and Vertical direction (since both AP and vertical directions are biphasic for any stride) and its inverse for medio-lateral direction (basic ML pattern is limb dependent and only repeated once for any given stride). A higher harmonic ratio represents a smoother walking pattern. 

Effects were considered significant when *p* < 0.05. We conducted initial analyses using a mixed-factor multivariate analysis of variance (MANOVA). Subsequent univariate repeated measures ANOVAs (mixed-factor design) were conducted separately for each dependent variable. Also, to control the familywise error rate Bonferroni corrections were adopted. 

## 3. Results

**Figure 7:**
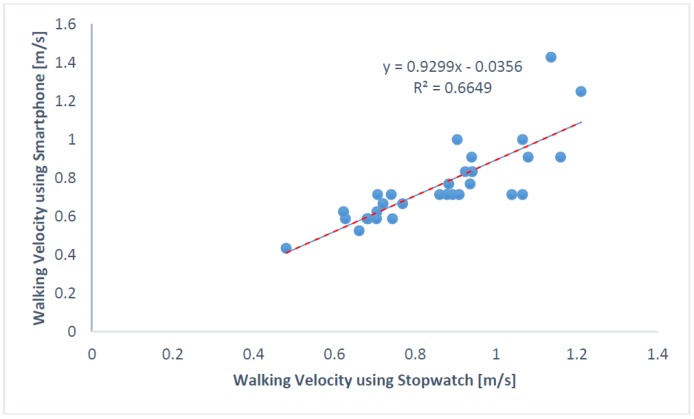
Relationship between the velocities of CVD patients computed from the two systems: stopwatch and smartphone. Dotted-red line shows the regression line.

Walking velocities computed using stopwatch time and smartphone time were found to be correlated with Pearson correlation coefficient = 0.8154 and spearman’s rho = 0.8834 (Figure 7). Eight participants were classified as frail and eight as non-frail using the velocities from the stopwatch (with cut-off velocity = 0.833 m/s).

**Figure 8:**
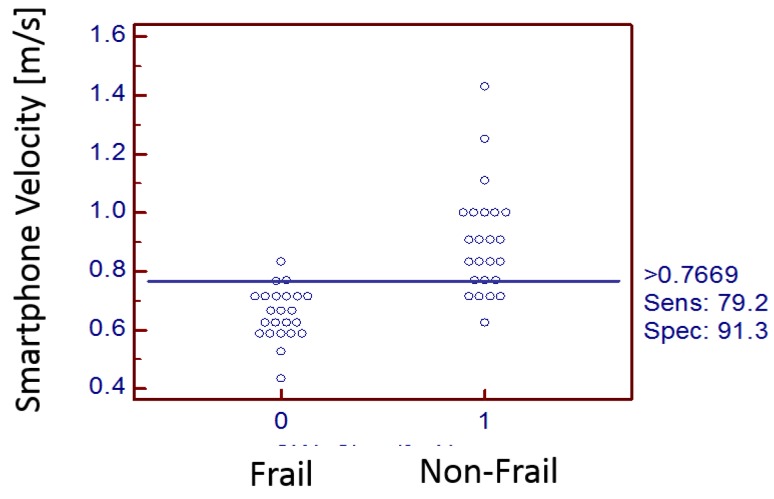
Integrative dot diagram suggesting specificity = 91.3% and Sensitivity = 79.2% for velocity derived from smartphone signals in classification of frail/non-frail patients.

Table 4 lists velocities from two different systems (stopwatch vs. smartphone). Figure 8 shows an interactive dot diagram of the data of the frail and non-frail groups as displayed in dots on two vertical axes. The horizontal line indicates the cut-off point with best separation (minimal false negative and false positive results) between the two groups. The specificity = 91.3% and sensitivity = 79.2% was found for the smartphone-based velocity predictions. The mean walking velocity measured using stopwatch for frail was 0.67 m/s and that for non-frail group was 0.98 m/s. However, smartphone sensors predicted the mean walking velocity for frail group as 0.75 m/s (corrected using the regression equation in Figure 7) and for non-frail group as 0.87 m/s (Table 5). Forceplate detected a significantly higher mean COP radius (*p* < 0.01), COP area (*p* < 0.01), COP path length (*p* < 0.01), mean COP Velocity (*p* < 0.01) and higher linear variability in parameters such as SD COP-AP (*p* < 0.01), SD COP-ML (*p* = 0.01), SD COP-R (*p* = 0.02). Complexity in AP direction, as defined by approximate entropy ApEn COP-AP, was found to be significantly lower in frail patients (*p* < 0.01). In congruence to this sample, entropy was also found to be lower in AP direction (*p* < 0.01) for the frail group. Mean power frequency in anterior-posterior direction was found to be lower in the frail group than that in the non-frail group (*p* < 0.01).

**Figure 9:**
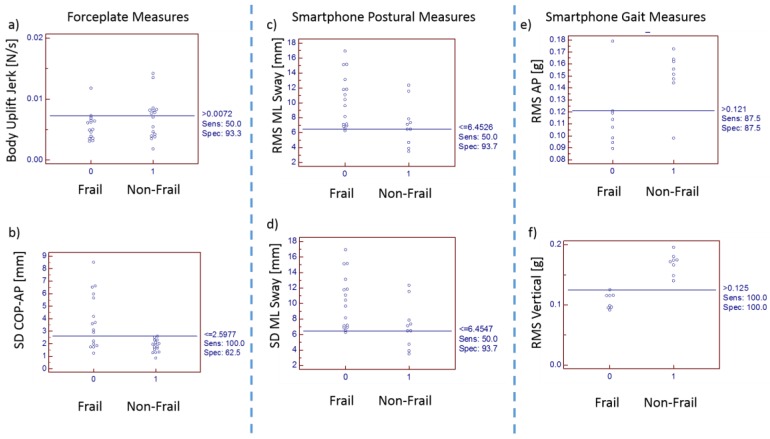
Interactive dot diagram of postural measures from forceplate: (**a**) Body uplift jerk; (**b**) SD of COP-AP, postural measures from smartphone; (**c**) RMS sway-ML; (**d**) SD of sway-ML and gait measures of smartphone; (**e**) RMS AP and (**f**) RMS Vertical.

Similar to the velocities measured using the stopwatch, it was found that smartphone-based walking velocity was significantly lower in frail patients than in non-frail patients (*p* < 0.01). Mean sway radius (*p* < 0.01), sway area (*p* < 0.01), sway path length (*p* < 0.05) and mean sway velocity (*p* < 0.05) were found to be significantly higher in frail patients than in non-frail patients (Figure 9).

**Figure 10:**
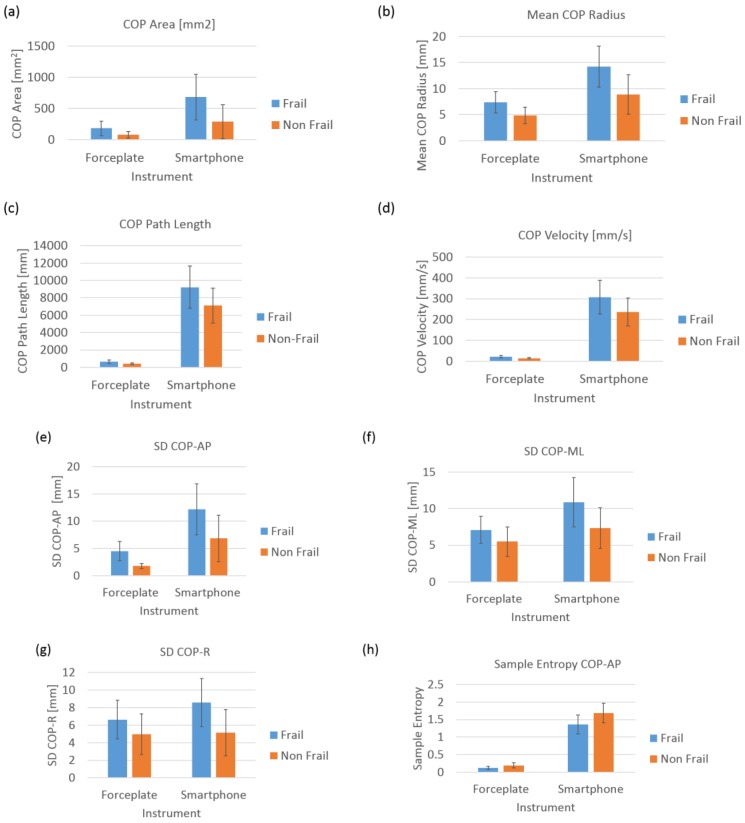
Postural parameters (**a**) COP Area; (**b**) Mean COP area; (**c**) COP path length; (**d**) COP Velocity; (**e**) SD of COP-AP; (**f**) SD of COP-ML; (**g**) SD of COP-R; (**h**) Sample Entropy of COP-AP from both systems i.e., forceplate and smartphone.

SD sway-AP (*p* < 0.01), SD sway-ML (*p* < 0.01), and SD sway-R (*p* < 0.01) were found to be significantly higher in frail participants. Similarly, RMS sway-AP (*p* < 0.01), RMS sway-ML (*p* < 0.01), RMS sway-R (*p* < 0.01) were also found to be significantly higher in frail participants (Figure 10). Complexity of sway signals in anterior-posterior direction measured by sample entropy SampEn AP (*p* < 0.01) and resultant sway direction SampEn R (*p* < 0.01) were found to be significantly lower than in the non-frail group.

Post-operative outcomes in CVD patients consisted of both morbidity and mortality. Two frail patients were diagnosed with stroke (ID06 and ID21), one frail patient (ID11) with renal failure, three frail patients (ID08, ID11 and ID21) were kept for prolonged ventilation, one frail patient (ID21) had to be re-operated, 3 frail (ID06, ID11 and ID22) and 1 non-frail (ID23) were sent to a skilled nursing facility, only one frail patient (ID11) had a length of stay more than 14 days, and one frail patient had mortality (ID21) (Table 6 and Table 7).

It was found that non-frail patients produced a higher range of accelerations while performing a sit-to-stand maneuver with lower overall variability (Table 8), whereas frail patients produced a lower range of accelerations while performing a sit-to-stand maneuver with higher variability (measured by Coefficient of variation). The variability in jerk produced during sit-to-stand was also found to be higher in frail patients than in non-frail patients. The mean time taken by frail patients in performing sit-to-stand and stand-to-sit was higher than non-frail patients (Table 8).

Root mean square (RMS) in all three directions was found to be significantly different in frail and non-frail patients. Non-frail patients produced significantly higher RMS-AP (*p* < 0.02), RMS-V (*p* < 0.01) and RMS-ML (*p* < 0.02) than frail patients (Table 9).

Interactive dot diagrams indicated that RMS in a vertical direction could provide results with 100% sensitivity and 100% specificity. Variability measures such as SD and RMS from smartphone postural stability data provided specificity of 93.7% and sensitivity of 50%. The cut-off point being 6.4547 mm could classify frailty with specificity of 93.7% (Figure 9 and Figure 10).

## 4. Discussion

Armed with the above-mentioned linear/nonlinear tools and inertial sensors for assessing movement variability, a trait of human movement performance, this study explored the smartphone sensor-based variables of variability in cardiovascular disease patients and their adverse post-operative outcomes. This study was conducted in a clinical environment using smartphone-based inertial sensors and found that variability of postural and gait movements in CVD patients was associated with frailty and adverse post-operative outcomes.

The Society of Thoracic Surgeons has recommended the use of quick tests such as gait speed for the assessment of frailty among cardiovascular patients. The frailty status in CVD patients is predictive of adverse health outcome, including falls, institutionalization, hospitalization and mortality [13,33,41,42]. Frail individuals are also at extremely high risk of falls, fractures and hospitalizations leading to death compared with their age-matched non-frail counterparts [13]. Gait speed suggested by the STS guideline is a robust measure in health care research, particularly among preoperative cardiac patients [43,44,45]. Therefore, all study patients were divided into a frail and non-frail group using the 5 m walk gait speed. This study has established a relationship between frail cardiac patients and their inherent variability and adverse post-operative outcomes after cardiac surgery. We have previously validated the use of inertial sensors in fall risk assessment in hemodialysis clinics [46,47]. Consistently, in this study, postural stability, postural transition times and gait speed (related to major health-related outcomes in frail population), are measured feasibly using smartphone inertial sensor-based methodology in clinical environments. There may be important losses of information when measurement of gait velocity is prone to human timing errors (use of stopwatch also requires experimenter’s attention and reaction time to press start or stop pushbuttons after visual verification of event). In clinical practice, where gait speed is an important predictive of severe health outcomes such as mortality and a subsequent physical disability, an objective, accurate, and reliable way is required for gait speed measurement. For this study, we devised the use of a smartphone with embedded inertial sensors which capture walking characteristics of patients in clinical environments. A five meter gait speed certainly does not introduce fatigue in patients with cardiovascular impairments awaiting surgery [45]. However, some patients who are awaiting their surgery also may not be healthy enough to walk for a 5 m distance. In such scenarios, it is worthwhile to examine the effects of postural control for balance and transitioning. Although it was found that in 5 m walking trials, that stopwatch time and smartphone time were highly correlated. However, it was found that smartphone-based frail classification had classified 3 non-frails erroneously as frails. Thus, if non-frail is the desired outcome with smartphone-based gait speed, the sensitivity = 79.2% and specificity = 91.3% when the cut-off velocity is chosen as 0.766 m/s rather than the prescribed 0.833 m/s. The feasibility and agreement of this smartphone app in estimation of 5 m gait speed in a clinical environment has been reported earlier with ICC(3,k) = 0.66 for normal walking speed in healthy adults. As expected, we found frail CVD patients (0.67 ± 0.08 m/s) walked slower than non-frail (0.98 ± 0.13 m/s) counterparts.

It was also found that frail CVD patients had increased fall risk as depicted by both linear and nonlinear measures of postural sway. In support of this hypothesis, we found a significant increase in linear parameters such as mean COP radius, COP area, COP path length, mean COP Velocity for frail patients than that in non-frail patients. Coherently, it was also seen in linear variability measures that frail patients had significantly higher standard deviation (SD) in anterior-posterior, medio-lateral and resultant directions of COP. Frail patients had significantly lower complexity than the non-frail patients in COP sway in anterior posterior direction. Statistical variability such as range and SD reflect overall magnitude of COP displacement, without considering the temporal structure of the COP time series. This fundamental difference may explain that nonlinear measures of postural signals reveal subtle temporal properties of signals which are not detected through traditional linear approach [48,49,50,51]. Traditionally, higher COP displacements have been linked with less stability and consequently, pathology. However, biological systems are intrinsically complex and linear analysis does not holistically account for the time-dependent evolution of the system, eschewing patterns within the time series and an appreciable amount of information on system dynamics. Thus, an increased COP movement may not unwittingly indicate deficient postural stability, but rather an element of a healthy vigilant system able to adapt to unexpected perturbations in an attempt to maintain balance.

Entropy-based estimations of signal irregularity and concurrent organizational variability represents the adaptive capacity of frail/non-frail participants to maintain balance. Frail participants were found to have significantly lower ApEn and SaEn values during prolonged quiet standing in the AP direction, indicating greater regularity and possibly decreased complexity. The findings coincide with previous investigations [52], linked with the theory of decreased complexity attributed to pathology and aging [53]. Probably, in frail patients the bodily degrees of freedom are constrained in the AP direction compared to the ML direction, whereby coordination of the physiological system, coupled with environmental interactions, lead behavioral processes into less complex, more stable response modes (i.e., more regular sway pattern and probably closed loop short-term dependencies to restore balance). Hence, the motor system is unable to adjust to the demands inherent to frailty, therefore movements transition to a more rigid postural control behavior in the AP direction—delineated by repeated patterns (high regularity) and decreased complexity–diminishing both adaptability and stability. In this context, the decrease in complexity may be due to impaired feedback control or strength, or impaired proprioception caused due to decreased physiological reserve in frail patients leading to reduced adaptive capacity of the underlying postural system [54].

Fractal analysis of the COP time series revealed relatively marginal differences in frail versus non-frail patients in all the AP and ML and resultant directions. From a biomechanics perspective, it may also be due to inability of elderly people to control and accelerate center-of-mass (COM) over base of support, perhaps due to lack of strength and degradation of type II fibers in skeletal muscles in presence of sarcopenia or any other frailty disorder. While muscle strength was not objectively measured in this study, it has been documented that many older people have relatively weaker tibialis anterior and vastus lateralis muscle strength compared to that of healthy adults [55,56]. Frailty is also found to be related with lower level of physical activity and impaired cardiorespiratory fitness and grip strength compared to lean counterparts [13], which could possibly impair an individual’s ability to correct a shift in the body’s COM and effectively prevent then from falling. Probably increased postural sway could be an adaptive strategy to provide additional stability under conditions of weakness in muscles involved for postural control. Age-related deterioration of sensory and neuromuscular control mechanisms could have added to this problem [57]. Degradation of balance shows that fall risk is increased in frail CVD patients. Smartphone-based variability information was found similar to that from forceplate; in addition, root mean square acceleration in AP, ML and resultant (R) directions were found to be significantly different among the frail CVD patients than the non-frail counter parts (Table 5). Additionally, significantly higher sample entropy was found in AP direction in frail patients using the smartphone.

Most of the frail patients were met with an adverse post-operative outcome which included stroke (2 frail), renal failure (1 frail), prolonged ventilation (3 frail), reoperation (1 frail), longer length of stay (1 frail) and admissions to skilled nursing facility (3 frail and 1 non-frail); there was one mortality of a frail patient. The post-operative outcomes such as stroke, renal failure, prolonged ventilation, reoperation, longer length of stay in intensive care, and admissions to skilled nursing facility can be classified as morbidity. The one non-frail elderly participant requested discharge to a skilled nursing facility due to personal/social reasons (in absence of anyone at home to take care of them).

Walking patterns and variability may be optimal from the perspective of energy expenditure [58], temporal variability [59], spatial variability [60], and attentional demands [61]. Stability while walking is important since up to 70% of falls occur during locomotion [62,63]. Moe-Nilssen evaluated walking stability using accelerometers at the lumbar spine [64,65], and reported higher average accelerations in people with balance impairments [66]. During the course of locomotion, humans respond to multiple irregular perturbations generated by walking. The task of maintaining stability while walking primarily requires controlling the motion of COM. It has been reported that normal subjects walking with plaster casts and crutches [40], amputees walking with prosthetic limbs [67] and older people with balance problems [68] have lower harmonic ratio. But we did not find any significant differences in harmonic ratios between the frail and non-frail group for 5 m distance walk in all 3 directions.

In this study, acceleration patterns were measured at the pelvis when walking (5 m walk) to provide an indicator of whole body’s stability in response to multiple unpredictable perturbations during walking. All humans have a preferable walking speed that is a combination of step length and step frequency and is an important factor in control of balance since, during walking, considerable potential for imbalance exists due to inertia of the upper body and the small contact area provided by the foot during a single limb stance [69]. This preferable or usual speed is selected to optimize the stability of the gait pattern. Hence, acceleration patterns were measured at the pelvis when walking to provide an indicator of whole body stability in response to multiple unpredictable perturbations during walking. However, we found that root mean square (RMS) in all three directions was significantly different in frail and non-frail patients. Probably, RMS acceleration is correlated with walking speed. The frail patient’s comfortable walking speed selected is slower when compared to non-frail patients to minimize acceleration variability but instead RMS values were found to be significantly higher and thus were unable to provide smooth and rhythmic movements of the pelvis. The interactive dot diagram suggested that postural measures from the forceplate such as jerk, SD of COP-AP, postural measures from the smartphone such as RMS sway-ML, SD of sway-ML and gait measures of the smartphone such as RMS AP and RMS Vertical are predictive of frailty with high accuracy.

These methods build on narrative descriptions of variability by quantifying qualities of postural control, postural transition and gait could serve as an indicator of surgery outcomes in CVD patients. In combination, linear and nonlinear variability analysis quantified postural and gait control to provide a more complete understanding of the adaptive strategies used in neuromuscular control than either method could provide alone. Thus, these inertial sensor-based variables are found to have high predictive validity to identify patients with adverse post-operative outcomes through an objective method of assessment using a smartphone. These fall risk indicators of variability could be used as prescreening tools for many different kinds of surgical procedures and in turn help clinicians to identify frail patients who may need intensive rehab or to preplan their stay in hospital with specialized nursing care before they return home.

The use of smartphones as medical devices has spread pervasively worldwide in the past decade. The scope of smartphone usage has certainly exceeded that initially envisioned for only telecommunication. The performance of smartphones depends on their different model as per their processing capability and embedded sensor quality. Undoubtedly, the quality of smartphone sensors is limited by sensor inaccuracy and imprecision [70,71]. It has been found that different smartphone models have different sensor-based bias, and sensor-based noise as quantified by Allan deviation in accelerometers as velocity random-walk (VRW) and angular random-walk (ARM) [72,73,74]. To eliminate noise and sensor bias differences dependent on various smartphone models, we have only used one smartphone (iPhone 5, Apple, Cupertino, CA, USA) for the entire experimental study. Thus, inertial sensors embedded inside the smartphone have the potential to measure gait and posture in CVD patients, although a great deal of work is required in future research to make such research tools easy to use for clinicians. The data collection was mainly conducted by hospital staff (Registered nurse specialist) using a smartphone and forceplate in the clinical setting and experimental protocols were modified as per clinical requirements. To meet the challenges of patient safety and point of care, new technologies are needed in future such that the patient data can be acquired without hindering medical routine for patients and hospital staff. 

### Limitations of the Study

The strength of the conclusions of this study must be tempered by the study’s limitations. The study population was limited to only 16 participants. The patients were aware that they were participating in a frailty assessment protocol. This could be a bias in the population we studied. They may be conscious of the environment and their performance may have been affected by the clinical environment (white coat syndrome). The hospital setting in which data were obtained for this study provided an unusual environment for the cardiac patients. We agree this is a special population who are battling for life and need help from physicians and researchers for their betterment. At the same time, they might be stressed to some extent for their surgery allotted for the next day and a non-laboratory setting limited the scope of this data. However, such analyses may provide insight as to the potential fall risk and chances of adverse post-operative outcomes were associated with the frail condition of patients.

Another limitation of the current study was that the smartphone-based assessments required patients to stand still before and after the 5-m walk. As automatic algorithms developed in the smartphone app determined velocity by evaluating start and stop times of movement. Automatic gait speed estimation by the smartphone required strict following of the protocol. If any other movement artifact is followed after or prior to the walking task, the movement time may get increased than the actual walking time and thus data had to be checked visually and truncated for correcting this.

## 5. Conclusions

The accurate measure of gait speed, as well as variability measures can improve the clinical evaluation of cardiac patients, providing an earlier detection of individuals at higher risk of major health-related events such as physical disability and mortality. This study demonstrated that a 5 m gait speed measurement using a smartphone is also a reliable objective measure; however, adhering to certain protocol is suggested for using a smartphone app. Although different methods have been used previously to measure gait speed and these have affected clinical interpretation and implementation of the gait speed [75,76]. By providing a smartphone-based clinically useful gait speed assessment method with a well-defined protocol which is simple, quick and easy to perform in clinics, it is hoped that using a smartphone for gait speed assessment will be promoted and encouraged in clinical and research settings. In addition, nonlinear postural variability measures such as complexity can be easily implemented in patients who are unable to walk but can stand still for at-least 30 s.

The study protocol and findings suggest that various variability parameters in walking and stand still posture can be easily implemented in cardiovascular clinical practice with high acceptability by the patients and clinical research staff. Patients started with standing still posture and walked at their usual pace, as if they were walking in their own home, and given no further encouragement or instructions. This data can be readily collected in non-laboratory environments and can be used to help interpret the results for future health-related events.

## Figures and Tables

**Table 1:** Means and standard deviations of patients’ anthropometric and age information.

Anthropometric Variables	Frail		Non-Frail		All CVD Patients	
	Mean	SD	Mean	SD	Mean	SD
Age (years)	76.38	4.03	76.00	3.55	76.18	3.67
Height (cm)	172.34	12.92	171.30	6.43	171.81	9.87
Weight (kg)	87.41	20.32	77.41	15.89	82.40	18.36
BMI (kg/m^2^)	29.69	7.46	26.26	4.73	27.97	6.28

**Table 2:** Voice commands used in app for data collection in clinical environment.

Time Interval (s)	Sound & Voice Commands	Total Elapsed Time (s)
0	Beep	0
10	Please Keep Sitting	10
10	Ready to Stand	20
10	Please stand	30
10	Very Good! Please Stand Still	40
65	Relax! Please Sit	105
60	Please Keep Sitting!	165
10	Ready to Stand	175
10	Very Good! Please Stand Still	185
65	Relax!	250

**Table 3:** Six variability parameters were calculated using accelerations from three directions.

Details	Parameter Computation	Equation Number
RMS vector magnitude	$\mathrm{RMST}=\sqrt{\mathrm{RMS}\_{\mathrm{AP}}^{2}+\mathrm{RMS}\_V^{2}+\mathrm{RMS}\_{\mathrm{ML}}^{2}},$	(2)
Normalized RMS in AP direction	${\mathrm{RMSR}}_{\mathrm{AP}}=\frac{{\mathrm{RMS}}_{\mathrm{AP}}}{\mathrm{RMST}},$	(3)
Normalized RMS in ML direction	${\mathrm{RMSR}}_{\mathrm{ML}}=\frac{{\mathrm{RMS}}_{\mathrm{ML}}}{\mathrm{RMST}}$	(4)
Normalized RMS in Vertical direction	${\mathrm{RMSR}}_{V}=\frac{{\mathrm{RMS}}_{V}}{\mathrm{RMST}},$	(5)
Harmonic Ratio in Vertical and AP direction	Harmonic Ratio=∑even harmonics/∑odd harmonics	(6)
Harmonic Ratio in ML direction	Harmonic Ratio=∑odd harmonics/∑even harmonics	(7)

**Table 4:** Classification of patients to frail and non-frail categories using velocities (cut-off = 0.833 m/s) from stopwatch. Velocities computed using smartphone walking signals are also listed below.

ID	Classification Frail (F), Non-Frail (NF)	Stopwatch Velocity			Smartphone Velocity			Classification Errors
		Mean	SD	CV	Mean	SD	CV	
ID04	NF	0.92	0.02	2.44	0.81	0.04	4.56	X
ID06	F	0.59	0.07	12.25	0.61	0.03	4.29	
ID08	F	0.73	0.05	6.44	0.66	0.06	9.70	
ID09	NF	1.21	0.07	5.89	1.23	0.22	17.56	
ID10	NF	1.06	0.14	12.95	0.97	0.05	5.41	
ID11	F	0.73	0.03	3.45	0.67	0.04	6.68	
ID13	F	0.83	0.09	10.27	0.75	0.07	9.11	
ID14	NF	1.06	0.02	2.34	0.81	0.16	20.38	X
ID17	F	0.67	0.01	1.51	0.58	0.05	8.60	
ID18	NF	0.91	0.03	2.97	0.70	0.07	10.36	X
ID19	F	0.58	0.09	15.04	0.59	0.14	24.14	
ID20	NF	0.97	0.10	9.92	0.88	0.04	4.95	
ID21	F	0.83	0.06	7.02	0.72	0.05	7.00	
ID22	F	0.74	0.05	6.95	0.65	0.10	16.11	
ID23	NF	0.95	0.09	9.24	0.77	0.06	7.71	
ID24	NF	1.00	0.06	6.33	1.01	0.10	10.04	

**Table 5:** Linear and non-linear variability parameters from forceplate and smartphone IMU’s for frail and non-frail participants.

Instrument	Variables	*p*-Value	Frail			Non-Frail		
			Mean	SD	CV	Mean	SD	CV
Stopwatch	Walking Velocity		0.67	0.08	12.03	0.98	0.13	13.10
Forceplate	Jerk [N/s]	0.83	0.01	0.00	72.96	0.01	0.00	46.55
	Peak-to-Peak Time [s]	0.09	1.81	0.86	47.33	1.38	0.69	49.71
	Mean COP Radius * [mm]	<0.01	7.38	2.02	27.30	4.88	1.59	32.60
	COP Area *	<0.01	183.37	114.36	62.36	82.44	53.97	65.46
	SD [COP-AP] *	<0.01	4.50	1.76	39.15	1.79	0.46	25.45
	SD [COP-ML] *	0.01	7.09	1.87	26.45	5.50	2.00	36.44
	SD [COP-R] *	0.02	6.63	2.20	33.17	4.98	2.33	46.87
	RMS [COP-AP]	0.92	28.61	25.42	88.87	27.62	36.61	132.51
	RMS [COP-ML]	0.08	55.13	32.29	58.57	40.29	18.68	46.36
	RMS [COP-R]	0.21	67.71	30.40	44.89	55.64	30.69	55.16
	Path Length [mm] *	<0.01	653.62	192.27	29.42	423.67	94.10	22.21
	Mean COP Velocity * [mm/s]	<0.01	21.79	6.41	29.42	14.12	3.14	22.21
	Alpha [COP-AP]	0.25	1.02	0.27	26.35	0.93	0.24	25.71
	Alpha [COP-ML]	0.13	0.93	0.20	21.40	1.03	0.24	22.85
	Alpha [COP-R]	0.20	0.92	0.21	22.31	1.01	0.24	23.47
	ApEn [COP-AP] *	<0.01	0.56	0.12	22.04	0.69	0.13	18.54
	ApEn [COP-ML]	0.25	0.74	0.15	20.58	0.69	0.14	19.83
	ApEn [COP-R]	0.28	0.71	0.17	23.49	0.66	0.12	18.56
	SampEn [COP-AP] *	<0.01	0.12	0.05	42.36	0.19	0.07	35.03
	SampEn [COP-ML]	0.30	0.19	0.09	47.49	0.16	0.07	41.49
	SampEn [COP-R]	0.34	0.18	0.09	51.19	0.16	0.05	34.15
	MPF [COP-AP] *	<0.01	0.03	0.01	35.61	0.04	0.02	35.92
	MPF [COP-ML]	0.10	0.04	0.02	44.30	0.04	0.01	32.56
Smartphone	Walk Velocity * [m/s]	<0.01	0.65	0.11	17.37	0.87	0.18	20.62
	Mean sway radius * [mm]	<0.01	14.24	3.93	27.59	8.88	3.76	42.36
	Sway Area * [mm^2^]	<0.01	682.54	362.45	53.10	289.02	270.77	93.69
	Path Length * [mm]	<0.05	9217.19	2426.75	26.33	7108.34	2010.22	28.28
	Mean sway Velocity * [mm/s]	<0.05	307.19	80.80	26.30	236.94	67.01	28.28
	SD [sway-AP] *	<0.01	12.18	4.69	38.55	6.87	4.27	62.10
	SD [sway-ML] *	<0.01	10.88	3.36	30.89	7.33	2.78	37.87
	SD [sway-R] *	<0.01	8.57	2.75	32.08	5.16	2.64	51.13
	RMS [sway-AP] *	<0.01	12.17	4.69	38.55	6.87	4.27	62.10
	RMS [sway-ML] *	<0.01	10.87	3.36	30.89	7.33	2.78	37.87
	RMS [sway-R] *	<0.01	16.65	4.67	28.05	10.29	4.54	44.16
	Alpha [sway-AP]	0.30	1.03	0.32	31.11	1.15	0.28	24.45
	Alpha [sway-ML]	0.54	1.10	0.24	21.60	1.05	0.20	18.61
	Alpha [sway-R]	0.49	0.96	0.23	24.00	0.90	0.21	23.78
	ApEn [sway-AP]	0.26	1.07	0.10	8.91	1.10	0.04	3.86
	ApEn [sway-ML]	0.43	1.09	0.05	4.91	1.08	0.08	7.76
	ApEn [sway-R]	0.40	1.14	0.05	3.95	1.13	0.03	3.10
	SampEn [sway-AP] *	<0.01	1.36	0.27	20.07	1.69	0.28	16.85
	SampEn [sway-ML]	0.08	1.24	0.25	19.78	1.42	0.31	21.96
	SampEn [sway-R] *	<0.01	1.48	0.22	14.98	1.70	0.20	11.55

* significant at *p* < 0.05.

**Table 6:** Post-operative morbidity and mortality of CVD patients.

ID	Frail/Non-Frail	Stroke	Renal Failure	Prolonged Ventilation	DSWI	Re-Operation > 24 h	Death	Skilled Nursing Facility	Length of Stay > 14 Days
ID04	NF	-	-	-	-	-	-	-	-
ID06	F	YES	-	-	-	-	-	YES	-
ID08	F	-	-	YES	-	-	-	-	-
ID09	NF	-	-	-	-	-	-	-	-
ID10	NF	-	-	-	-	-	-	-	-
ID11	F	-	YES	YES	-	-	-	YES	YES
ID13	F	-	-	-	-	-	-	-	-
ID14	NF	-	-	-	-	-	-	-	-
ID17	F	-	-	-	-	-	-	-	-
ID18	NF	-	-	-	-	-	-	-	-
ID19	F	-	-	-	-	-	-	-	-
ID20	NF	-	-	-	-	-	-	-	-
ID21	F	YES	-	YES	-	YES	YES	N/A	N/A
ID22	F	-	-	-	-	-	-	YES	-
ID23	NF	-	-	-	-	-	-	YES	-
ID24	NF	-	-	-	-	-	-	-	-

**Table 7:** Definitions of criteria for morbidity and mortality.

Stroke	Stroke (Central Neurological Deficit Persisting > 72 h)
RF	Renal Failure (new requirement for dialysis or increase in serum creatinine > 153 micromoles/liter [>2 mg/dL] and >2-fold the preoperative level)
Vent	Prolonged Ventilation (>24 h)
DSWI	Deep Sternal Wound Infection (requirement for operative intervention and antibiotic therapy, with positive culture)
Reop	Need for reoperation (for any reason)
SNF	Discharge to skilled Nursing Facility (health care facility) (rehabilitation, Convalescence, other hospital, nursing home) for on-going care or rehabilitation
LOS	Prolonged length of stay (>14 days postoperatively)
Death	Death (all causes)

**Table 8:** Variabilities in sit-to-stand and stand-to-sit movement parameters in frail and non-frail elderly CVD patients.

Postural Transition Variables	Frail						Non-Frail					
	Sit to Stand			Stand to Sit			Sit to Stand			Stand to Sit		
	Mean	SD	CV	Mean	SD	CV	Mean	SD	CV	Mean	SD	CV
Acc Range (m/s^2^)	44.6	23.8	53.4	55.3	23.0	41.6	52.4	25.0	47.7	72.6	31.3	43.0
Jerk (mm/s^3)^	15.8	13.9	87.8	17.3	12.4	71.5	15.9	9.2	58.1	21.6	11.1	51.4
Time (s)	1.6	0.6	41.8	1.5	0.7	48.0	1.4	0.4	32.4	1.3	0.3	23.1

**Table 9:** Harmonic ratio and root mean square (RMS-AP, ML, V) and normalized RMSR-AP, ML and V from 5 m walk smartphone signals.

Variables	Health Status					
	Frail			Non-Frail		
	Mean	SD	CV	Mean	SD	CV
Harmonic Ratio_AP	1.06	0.17	15.96	1.16	0.19	16.77
Harmonic Ratio_V	0.96	0.29	30.50	1.18	0.26	22.14
Harmonic Ratio_ML	1.09	0.37	34.36	0.98	0.25	25.19
RMS_AP *	0.12	0.03	24.50	0.15	0.02	15.23
RMS_V *	0.11	0.01	11.82	0.17	0.02	10.40
RMS_ML *	0.11	0.02	19.63	0.15	0.03	22.48
RMSR_AP	0.59	0.07	11.57	0.55	0.09	16.90
RMSR_V	0.56	0.07	12.89	0.62	0.05	8.47
RMSR_ML	0.58	0.05	9.41	0.55	0.09	15.50

* significant at *p* < 0.05.
